# Signaling Specificity Provided by the *Arabidopsis thaliana* Heterotrimeric G-Protein γ Subunits AGG1 and AGG2 Is Partially but Not Exclusively Provided through Transcriptional Regulation

**DOI:** 10.1371/journal.pone.0058503

**Published:** 2013-03-08

**Authors:** Leena Thung, David Chakravorty, Yuri Trusov, Alan M. Jones, José Ramón Botella

**Affiliations:** 1 Plant Genetic Engineering Laboratory, School of Agriculture and Food Sciences, University of Queensland, Brisbane, Queensland, Australia; 2 Departments of Biology and Pharmacology, University of North Carolina at Chapel Hill, Chapel Hill, North Carolina, United States of America; Ghent University, Belgium

## Abstract

The heterotrimeric G-protein complex in *Arabidopsis thaliana* consists of one α, one ß and three γ subunits. While two of the γ subunits, AGG1 and AGG2 have been shown to provide functional selectivity to the Gßγ dimer in Arabidopsis, it is unclear if such selectivity is embedded in their molecular structures or conferred by the different expression patterns observed in both subunits. In order to study the molecular basis for such selectivity we tested genetic complementation of AGG1- and AGG2 driven by the respectively swapped gene promoters. When expressed in the same tissues as AGG1, AGG2 rescues some *agg1* mutant phenotypes such as the hypersensitivity to *Fusarium oxysporum* and D-mannitol as well as the altered levels of lateral roots, but does not rescue the early flowering phenotype. Similarly, AGG1 when expressed in the same tissues as AGG2 rescues the osmotic stress and lateral-root phenotypes observed in *agg2* mutants but failed to rescue the heat-stress induction of flowering. The fact that AGG1 and AGG2 are functionally interchangeable in some pathways implies that, at least for those pathways, signaling specificity resides in the distinctive spatiotemporal expression patterns exhibited by each γ subunit. On the other hand, the lack of complementation for some phenotypes indicates that there are pathways in which signaling specificity is provided by differences in the primary AGG1 and AGG2 amino acid sequences.

## Introduction

Heterotrimeric G-proteins (G-proteins), consisting of three subunits Gα, Gß and Gγ, are involved in a diverse range of vital biological processes including hormone regulation, neurotransmission, light perception and cell proliferation [1], [2], [3]. In animal systems, G-proteins mediate signaling initiated by seven transmembrane (7 TM) spanning G-protein coupled receptors (GPCRs) after activation by an external stimulus. Activation of the GPCR promotes the exchange of GDP for GTP in the Gα subunit and as a result Gα-GTP dissociates from the Gßγ dimer, allowing Gα-GTP and Gßγ to activate their respective downstream effectors. Termination of signaling occurs when GTP is hydrolyzed to GDP by the intrinsic GTPase activity of Gα and the inactive heterotrimer re-associates back at the receptor [4]. In plants, the G protein complex is self-activating [5], [6], [7], [8] and therefore does not need a GPCR [9]. Instead, many plants utilize a 7 TM Regulator of G Signaling (RGS) protein, that regulates the GTP hydrolysis reaction of the Gα subunit (Urano. unpublished).

In humans, there are 23 Gα, 5 Gß and 12 Gγ subunits, allowing a large number of different heterotrimer combinations Gα_x_ß_y_γ_z_ [10], [11], [12], [13], [14]. It is generally believed that the diversity of heterotrimer combinations provides the required coupling specificity to allow signaling by over 800 GPCRs [15]. It was initially thought Gα was the only subunit active in signaling relegating the role of the Gßγ dimer to Gα inactivation and escort to the receptor. It was subsequently established that different Gßγ dimers interact with specific effectors, proving that the Gßγ dimer contributes to both active signaling and heterotrimer specificity [16], [17]. Signaling specificity by the different Gßγ dimers can be a consequence of the dimer’s intrinsic structural properties but it can also be dictated by the tissue specificity of their expression patterns [18], [19], [20], [21], [22].

Plant G-proteins are also involved in numerous signaling processes including interactions with rhizobia [23], defense against pathogens [24], [25], [26], [27], [28], [29], [30], morphological development and growth [31], [32], [33], cell proliferation [34], [35], ion-channel regulation [36], stomatal control [37], [38], light perception [32], [39], [40], [41], [42], abiotic stress [43], [44], [45], [46] and hormonal responses including glucose, brassinosteroid, abscisic acid and jasmonate [47], [48], [49], [50], [51], [52], [53]. G-proteins have also been linked to yield related quantitative trait loci in important crops such as rice [54], [55]. Unlike animals, where G-proteins underwent extensive subunit duplication and divergence of function, the plant G-protein repertoire is much simpler. Only one Gα (GPA1), one Gß (AGB1) and three Gγ subunits (AGG1, AGG2 and AGG3) are encoded in the *Arabidopsis thaliana* genome [54], [56], [57], [58], [59].

Among the three Arabidopsis Gγ subunits, AGG1 (Gγ1) and AGG2 (Gγ2) strongly resemble the canonical mammalian Gγ [58], [59], [60]. AGG3 (Gγ3) on the other hand is quite different from AGG1 and AGG2 being more than twice the size (253 a.a.) and exhibiting a modular structure with a γ-like domain at its N terminus, followed possibly by a transmembrane domain and a long cysteine rich C-terminal region [29], [54], [58], [59]. Despite AGG1 and AGG2 sharing extensive sequence conservation (48% amino acid identity and 65% similarity considering conservative substitutions), Trusov et al. [61] reported that Arabidopsis *agg1* and *agg2* mutants exhibit distinct phenotypes, prompting the hypothesis that the different Gγ subunits confer specificity to the Gßγ dimer in plants. An important and still unanswered question is the molecular basis for such specificity. In normal circumstances it would be fair to assume that the basis for the specificity resides in the molecular structure of the two Gγ subunits (and ultimately in their amino acid composition). Nevertheless, promoter studies showed that the two closely related AGG1 and AGG2 subunits have tissue and developmental expression patterns that rarely overlap [61], [62], raising the possibility that the basis for the specificity could be either partially or totally provided by their mutually-exclusive expression patterns. In leaves, AGG1 expression was restricted to veins, while AGG2 expression was observed primarily in guard cells. In roots AGG1 expression was restricted to the stele while AGG2 expression was excluded from the stele yet found in the cortex and epidermis. This would provide a transcriptional means to control the level of the Gßγ subunit on the plasma membrane and therefore the capacity for signal output.

A number of hypothetical scenarios can be envisaged including (i) Gßγ1 and Gßγ2 may activate specific sets of effectors, therefore mediating different signaling processes; (ii) Gßγ1 and Gßγ2 may activate common sets of effectors, with their presence or absence in an individual tissue dictating their involvement in signaling and (iii) an intermediate case in which spatio-temporal separation of expression and a degree of effector specificity contribute to the final response.

In order to determine if the specificity observed for Gγ function resides in transcriptional control of the *AGG1* and *AGG2* genes we swapped gene promoters and tested for genetic complementation in the respective Arabidopsis *agg1* and *agg2* mutants. Phenotypic analyses revealed that AGG1 and AGG2 are able to complement some but not all mutant phenotypes, indicating the existence of both transcriptional spatial and temporal regulation of the Gßγ activity but also suggesting that some signaling specificity information resides in the primary amino acid composition of both subunits.

## Materials and Methods

### Plant Material

The *agg1-1c* mutant allele of *AGG1* (At3g63420) and the *agg2-1* mutant allele of *AGG2* (At3g22942) in the Col-0 background, were described previously [61].

To generate the *agg1-1c AGG1:AGG1* complementation lines, an *AGG1* fragment from ∼2 kb 5′ of the start codon to ∼0.8 kb 3′ of the stop codon was amplified from wild-type genomic DNA using Elongase (Invitrogen). The primers used were: 5′-GAAAGAGAGGTCTGGTTAGCTATGC-3′ and 5′-GAAGGAGCTCTAATGAGGTCATCAAC-3′. The resulting 3.8 kb fragment was cloned into the pGEM-T Easy vector (Promega) and transferred using *Eco*RI sites into the binary vector pCAMBIA1380. Subsequently, the construct was transformed into *Arabidopsis agg1-1c* plants by *Agrobacterium tumefaciens*–mediated transformation [63]. Primary transformants were selected with hygromycin B. At least ten independent homozygous transgenic lines were obtained.

The *agg1-1c AGG1:AGG2* transgenic Arabidopsis were generated as follows. Elongase (Invitrogen) was used to amplify sequences from wild-type Arabidopsis genomic DNA. The following primers were used: for the *AGG1* promoter region, 5′-GGGGTACCGCGGCCGCTGATGAGACACACAATCAAAC-3′ and 5′-GGCTCGAGTCTCGCTAGCAGGTCGCA-3′; for the coding region and terminator of *AGG2,* 5′-GGCTCGAGTGATGGAAGCGGGTAGCTC-3′ and 5′-GCGGCCGCGTTTTGGTTCATGATGTTTCCT-3′. Restriction sites (underlined) were incorporated on the ends of fragment for cloning purposes. The PCR products were ligated into pGEM-T Easy vector (Promega). The *AGG2* fragment was transferred into the pBluescript SK+ vector using *Xho*I and *Not*I restriction sites. *AGG1* promoter fragment was inserted in front of *AGG2* fragment in the pBluescript SK+ using *Kpn*I and *Xho*I restriction sites. PCR was then performed on the AGG1 promoter-AGG2-pBluescript SK+ construct with the following primers: 5′-GGGGACAAGTTTGTACAAAAAAGCAGGCTGTAAAACGACGGCCAG-3′ and 5′-GGGGACCACTTTGTACAAGAAAGCTGGGTCAGGAAACAGCTATGAC-3′, in order to flank A*GG1p*::*AGG2* fragment with *att*B1 and *att*B2 gateway recombination sequences. The *att*B1-A*GG1p*::*AGG2-att*B2 PCR product was recombined into pDONR™207 using Gateway® BP Clonase® (Invitrogen). A reaction was performed with Gateway® LR Clonase® system to clone A*GG1p*::*AGG2* into the binary vector pMDC99. The construct was again transformed into *agg1-1c* by the floral dip method. Transformants were selected based on resistance to hygromycin B.

*agg2-1 AGG2p::AGG2* transgenic lines were generated by transformation of a 4.7 kb *AGG2* fragment, from1.6 kb 5′ of the start codon to 1.8 kb 3′ of the stop codon, into *agg2-1*. The primers used to amplify the 4.7 kb fragment were 5′-GGTACCGCGGCCGCATTGCCAGCCGATTTTTGCC-3′ and 5′-GCGGCCGCGTTTTGGTTCATGATGTTTCCT-3′. The resulting fragment was cloned into pGEM-T Easy (Promega),and transferred to the binary vector pUQC477 using terminal *Not*I restriction sites. The final construct was transformed into *agg2-1* by floral dip, and transformants were selected using BASTA as described elsewhere [64].

The *agg2-1 AGG2p::AGG1* mutant lines were generated as follows. The *AGG2* promoter was amplified using the primers 5′-GGTACCGCGGCCGCATTGCCAGCCGATTTTTGCC-3′ and 5′-GGCTCGAGAAATTTCTCGAATTCAACCCTC-3′. The *AGG1* coding region and terminator were amplified with 5′-GGCTCGAGGGATGCGAGAGGAAACTGT-3′ and 5′-GGGCGGCCGCTTTAACGGCTAACTTACTTATC-3′. The resulting two fragments were each ligated into pGEMT-Easy (Promega). The *AGG1* coding region and terminator fragment was then transferred into pBluescript SK+ vector using *Xho*I and *Not*I restriction sites. The *AGG2* promoter fragment was inserted in front of the *AGG1* fragment using *Kpn*I and *Xho*I restriction sites. The *AGG2p::AGG1* fragment was then transferred into the pUQC227 vector using terminal *Not*I restriction sites. The final construct was transformed into *agg2-1* by the floral dip method, and transformants were selected using BASTA.

### Quantitative Real-time RT-PCR Analysis

Total RNA was extracted from two-week-old seedlings as described previously [65]. First strand cDNA synthesis was conducted using the SuperScript III RT kit (Invitrogen) according to the manufacturer’s instructions. qRT-PCR was performed using Power SYBR Green PCR Master Mix (Applied Biosystems) and the 7900 HT Sequence Detection System (Applied Biosystems). The following primer pairs, designed using Primer Express software (Applied Biosystem), were used in the qRT-PCR: *AGG1* 5′UTR, forward, 5′-GAGAGAGACTTCGACGACAATTCA-3′, reverse, 5′-CTCGCTAGCAGGTCGCAGAT-3′; *AGG1* exon2, forward, 5′- GGAGGTCGAGAACACAGATATTGTATC-3′, *AGG1* exon3, reverse, 5′- CAACAGAGGATCGGGTCCTTT-3′; *AGG1/AGG2*, forward, 5′- TGCGACCTGCTAGCGAGACT-3′, reverse, 5′- CCTGTGTTTGCCTCTTGTATCAAC-3′; *AGG2* 5′ UTR, forward, 5′- CCCCAACTCATAACTTTGAATTTTCTA-3′, reverse, 5′- GGATTCAGAATCAAACAGATCTTGAGA-3′; *AGG2* exon3, forward, 5′- GCATCAGCATCCTGCAAAGA-3′, reverse, 5′- GGACCTGTTGTTTCGGGAAGA-3′; *AGG2/AGG1*, forward, 5′- GTTTCGATTTTTATTTTGAGGGTTGA-3′, reverse, 5′- CCCGCCGTGAGAAACAGA-3′. The previously validated *β-ACTIN2*, *β-ACTIN7* and *β-ACTIN8* were used as reference genes to quantify relative expression [66]. Gene expression analysis was performed using SDS Version 2.2.2 software (Applied Biosystems). The results were average values from three independently prepared RNA samples.

### Mutant Characterization

Plants were grown under a long-day conditions (16 h light/8 h dark) with cool white fluorescent bulbs at approximately 100 µmol m^−2^ s^−1^ and 22°C unless stated otherwise. All statistical analysis was performed with the GraphPad Prism version 5.04 for Windows (GraphPad Software, San Diego California USA, www.graphpad.com). All experiments were repeated at least three times with similar results.

### Fusarium Culture Preparation and Inoculation

*Fusarium oxysporum* f. sp. *conglutinans* (BRIP 5176, Department of Primary Industries, http://www.dpi.vic.gov.au, Queensland, Australia) culture preparation and root inoculations were performed as previously described [67] with modifications. Briefly, *F. oxysporum* was grown for approximately 1 week on one-half-strength potato dextrose agar plates at 25°C. Two plugs were cut from these plates under sterile conditions and placed into a flask containing 250 mL of potato dextrose broth. The flask containing the inoculum was then grown for approximately 3 days at 28°C with shaking at 110 rpm. The culture was filtered through Miracloth (Calbiochem, San Diego) and quantified with a hemocytometer. The suspension was diluted with sterile distilled water to a concentration of 0.5 x 10^6^ spores mL^–1^. Two-week-old plants which had been grown on steam sterilized soil were used for the assay. Before inoculation, the plants were carefully removed from the soil, the roots rinsed with water and dipped for at least 30 seconds into the fungal inoculum. The inoculated plants were replanted into fresh soil and grown at 27°C. Twenty plants from each of the wild-type and mutant lines were inoculated in two independent inoculation experiments. The degree of infection were scored as symptoms appeared and progressed in the window of days 7 to 12 post-inoculation, by counting the number of yellow and dead leaves as a percentage of the total number of leaves [66].

### Flowering time Analysis

Seeds were sown on soil and stratified for 48 hours at 4°C in darkness. Thirty plants per line were grown at 22°C. Where flowering induction is required, seedlings were initially grown at 22°C for two weeks before being transferred to a 29°C growth room. Flowering time was determined by the age of the plant in days when the inflorescence reached approximately 1 cm in height from the rosette.

### Plate Assays

All plates contained 0.5X MS basal salts (PhytoTechnology Laboratories), 0.8% phytagel (Sigma), and varying amount of sucrose [68]. No sucrose was added to plates used for germination assays. Seeds were dry sterilized by 4 hour incubation in a chamber filled with chlorine gas. After sowing onto solid media, all seeds were stratified for 48 h at 4°C in darkness. 6% w/v D-mannitol was added to the plates used for the osmotic stress germination assay. Germination was determined as an obvious protrusion of the radicle. For root assays, seedlings were grown at 26°C on vertical plates supplemented with 1% sucrose for 14 days, and the number of lateral roots per seedling was counted using a dissecting microscope. For adventitious root induction, media containing 3% sucrose was autoclaved. Once cooled to 55°C, NAA was added to a final concentration of 1 µM, from a stock solution of 10 mM. Hypocotyls from 5-day-old etiolated seedlings were aseptically excised and transferred onto the NAA supplemented media. Adventitious root development on the plate was photographed after 10 days incubation at 26°C.

## Results

### Complementation Constructs and Transgene Expression

**Figure 1:**
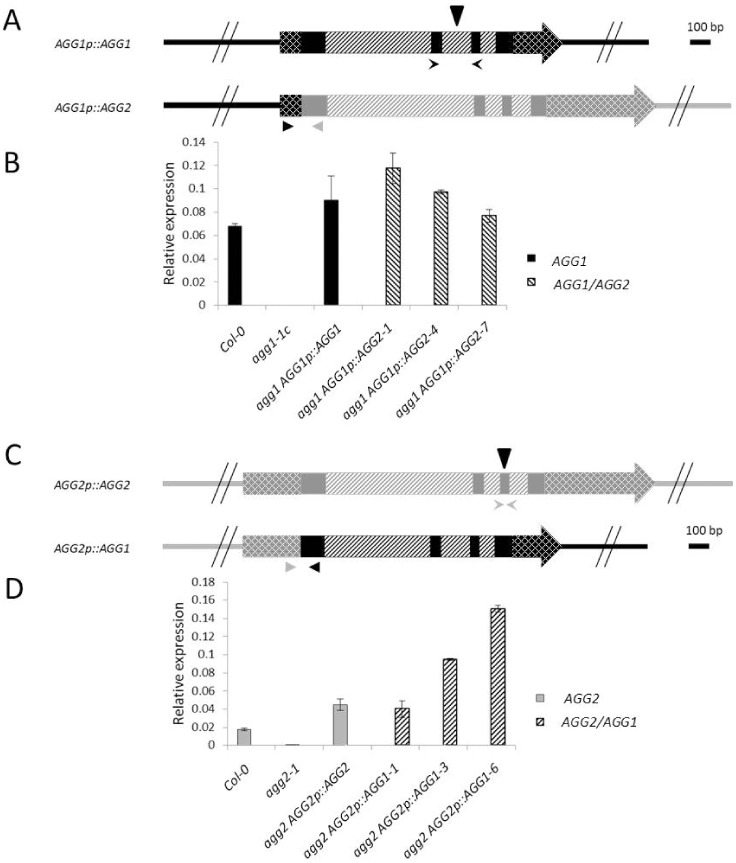
*AGG1* and *AGG2* complementation constructs and expression analysis in transgenic lines. (A) *AGG1* complementation constructs. The *AGG1p::AGG1* construct contains the entire *AGG1* gene starting 1 kb upstream of the 3′ untranslated region (UTR) and extending 1.8 kb downstream of the 3′ UTR region to include the native *AGG1* termination sequence. The *AGG1p::AGG2* construct contains the *AGG1* promoter region as well as the *AGG1* 5′ UTR, fused to an *AGG2* genomic fragment, starting at the ATG start codon and including 1.8 kb downstream of the 3′ UTR to include the native *AGG2* termination sequence. Expression of the *AGG1p::AGG2* construct will result in a ‘hybrid’ mRNA molecule containing the 5′UTR from *AGG1* fused to the coding region from *AGG2*. (B) Relative mRNA expression levels. Solid black bars show the relative abundance of the *AGG1* mRNA in WT (Col-0) and *agg1-1c* mutant plants. Self-complementation of the *agg1-1c* mutant with the *AGG1p::AGG1* construct results in similar levels of *AGG1* mRNA to those observed in WT plants. Cross-complementation of the *agg1-1c* mutant with the *AGG1p::AGG2* construct results in similar levels of expression of the *AGG2*hybrid mRNA (dashed bars) to those observed for *AGG1* mRNA in WT and self-complementation lines. (C) *AGG2* complementation constructs. The *AGG2p::AGG2* construct contains the entire *AGG2* gene starting 2 kb upstream of the 3′ untranslated region (UTR) and extending 1.8 kb downstream of the 3′ UTR region to include the native *AGG2* termination sequence. The *AGG2p::AGG1* construct contains the *AGG2* promoter region as well as the *AGG2* 5′ UTR, fused to an *AGG1* genomic fragment, starting at the ATG start codon and including 1.8 kb downstream of the 3′ UTR to include the native *AGG1* termination sequence. Expression of the *AGG2p::AGG1* construct will result in a ‘hybrid’ mRNA molecule containing the 5′UTR from *AGG2* fused to the coding region from *AGG1*. (D) Relative mRNA expression levels. Solid grey bars show the relative abundance of the *AGG2* mRNA in WT (Col-0) and *agg2-1* mutant plants. Self-complementation of the *agg2-1* mutant with the *AGG2p::AGG2* construct results in similar or higher levels of *AGG2* mRNA to those observed in WT plants. Cross-complementation of the *agg2-1* mutant with the *AGG2p::AGG1* construct results in equal or higher levels of expression of the *AGG1* hybrid mRNA (dashed bars) to those observed for *AGG2* mRNA in WT and self-complementation lines. In (A) and (C), *AGG1* genomic sequences are represented in black while *AGG2* sequences are in grey. Regions upstream of the 5′UTRs (promoters) and downstream of the 3′ UTRs (terminators) are represented in solid lines; 5′ and 3′ UTRs are represented in dashed boxes, exons are represented in solid boxes and introns are represented in white boxes. Arrows represent the position of the primers used for real time quantitative PCR (RT-qPCR). The solid triangles show the position of the T-DNA insertions in the *agg1-1c* and *agg2-1* mutants. In (B) and (D), transcript levels are shown as relative to *ACTIN* genes expression, mean ± SE of three replicas.

To determine whether Gγ1 and Gγ2 are functionally interchangeable we designed a cross-complementation strategy. Since the expression profiles of *AGG1* and *AGG2* are non-overlapping we carefully designed the complementation constructs trying to reproduce as much as possible the genomic environment for each of the two genes. In order to complement the *agg1* mutation, we fused 1 kb of the promoter region of *AGG1* (including the 5′ UTR) to an *AGG2* genomic fragment containing the entire gene, starting at the start ATG codon and ending 1.8 kb downstream of the 3′ UTR to include the terminator sequences (AGG1p::AGG2; Fig. 1A). To complement the *agg2* mutation we fused 2 kb of the *AGG2* promoter region, including the *AGG2* 5′ UTR, to a genomic fragment containing the entire *AGG1* gene from the translational start codon and extending 1.8 kb downstream of the 3′ UTR (*AGG2p::AGG1*; Fig. 1C). As positive controls we prepared constructs containing the entire *AGG1* and *AGG2* genes, including promoter and terminator regions (*AGG1p::AGG1* and *AGG2p::AGG2* respectively in Fig. 1A and 1C).

**Figure 2:**
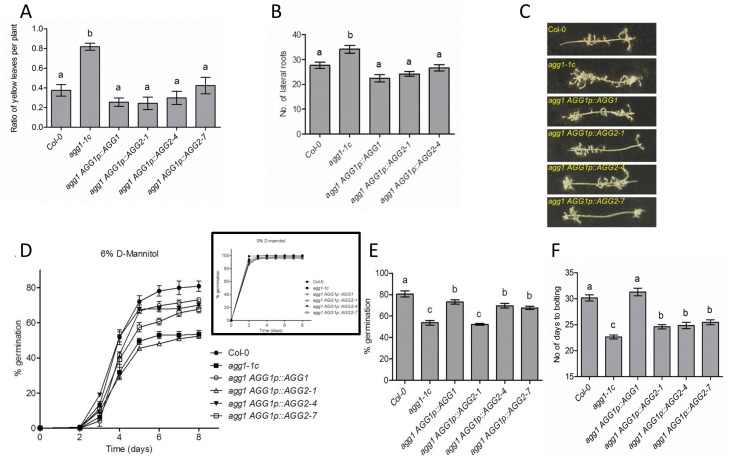
AGG2 complements some but not all *agg1* mutant phenotypes. (A) Sensitivity to *F. oxysporum*. Roots of two-week-old seedlings were inoculated with *F. oxysporum* spores and total number and number of chlorotic leaves were counted 9 days after inoculation for each plant. The ratio of chlorotic/total number of leaves was used to evaluate disease progression in infected plants. Bars on the graph represent average values estimated for 20 plants per each genotype. Error bars show standard errors. Letters indicate groups with statistically significant differences in disease progression (P<0.05, one-way ANOVA). (B) Total number of lateral roots was scored in two-week-old seedlings grown vertically on 0.5x MS supplemented with 1% sucrose. Bars represent average values ±SE of 15 plants per genotype. Letters indicate groups with statistically significant differences in number of lateral roots (P<0.05, one-way ANOVA). (C) Adventitious root development in excised hypocotyls was induced by supplementing media with 1µM NAA. Photos of representative hypocotyls from each tested genotype are shown. (D) Germination dynamics of wild-type, mutant and complementation lines grown on 0.5x MS supplemented with 6% D-mannitol during 8 days after stratification. Each genotype was analyzed in three replica plates with more than 100 seeds. Insert shows control germination without D-mannitol. (E) Percentage of germinated seeds at day 8 from panel (D) showing the highest difference between genotypes. Bars represent average value of three replicates (more than 100 seeds each). Error bars show standard errors. Letters indicate groups with statistically significant differences in seed germination (P<0.05, one-way ANOVA). (F) AGG2 partial rescue of the early flowering phenotype observed in *agg1-1c* mutants. Plants were grown under long day conditions (16 h light/8 h dark) at 23°C. Day of inflorescence appearance was recorded for at least 30 plants of each genotype. Bars represent average number of days from germination till inflorescence appearance ± SE. Letters indicate groups with statistically significant differences (P<0.05, one-way ANOVA).

The *AGG1p::AGG2* and *AGG1p::AGG1* constructs were used to obtain transgenic Arabidopsis lines in the *agg1-1c* T-DNA mutant background (designated *agg1 AGG1p::AGG2* and *agg1 AGG1p::AGG1* respectively in this work) while the *AGG2p::AGG1* and *AGG2p::AGG2* were introduced into an *agg2-1* background (designated *agg2 AGG2p::AGG1* and *agg2 AGG2p::AGG2* respectively, in this work) [61]. At least ten homozygous transgenic lines were generated from each of the constructs. Expression of the transgenes was analyzed in all transgenic lines and those with silencing or aberrant expression, as well as those with obvious insertional effects were discarded. Three lines for each of the complementation constructs and one line for each control were further characterized. The length of the promoter regions used in this study, especially in the case of *AGG1*, was chosen to maximize the length of the upstream region for each gene without including the full coding region of the neighboring genes which may lead to unwanted ectopic effects. The ultimate proof that we captured the entire promoter is the observation that the promoter segments chosen were able to drive genetic complementation of the cognate coding sequence. For example, the chosen *AGG1* promoter segment driving expression of the *AGG1* coding region genetically complemented the *agg1* mutant (Figure 2).

To test the hypothesis that transcriptional regulation imparted at least part of the functional specificity of the Gγ subunits we expressed a hybrid messenger RNA containing the 5′UTR of *AGG1* fused to the coding region and 3′UTR of *AGG2*. This was intentionally designed to include possible *AGG1* regulatory elements present in this region, which could influence transcription or translation rates, and to account for any influence of this region in the stability of the mRNA. This strategy also provided a robust and reliable method to quantify the expression of the transgene using quantitative real time PCR with a forward primer located in the 5′UTR of *AGG1* and a reverse primer located in the coding region of *AGG2*. This combination of primers detects only hybrid RNA molecules avoiding detection of the native *AGG2* mRNA and any incomplete or aberrant *AGG1* mRNA present in the *agg1-1c* T-DNA mutant.

A critical pre-requisite for the success of our approach is to obtain transgenic lines with transgene expression levels at least equal to those observed for the native gene in wild type plants. Fig. 1 shows the relative expression levels determined using Quantitative RT-PCR for all genotypes used in this study (wild-type, mutant and transgenic complementation lines). The *agg1 AGG1p::AGG1* complementation line produced similar AGG1 transcript levels to wild-type plants while the levels of the *AGG2* hybrid transcript in the *AGG1p::AGG2* cross-complementation lines were also similar to wild-type AGG1 levels (Fig. 1B). As expected, no AGG1 transcript was detected in the agg1-1c mutant. Conversely, the *AGG2p::AGG1* construct generates a hybrid messenger RNA containing the 5′UTR of *AGG2* fused to the coding region and 3′UTR of *AGG1*. Analysis of the *agg2* self- and cross-complementation lines showed transcript levels equal or greater to wild-type levels (Fig. 1D). In order for the cross-complementation strategy to work it was important to achieve at least the same levels of expression present in wild type plants, therefore higher expression levels in the transgenic lines, compared to wild type plants was still useful to determine if AGG1 complements *agg2* mutants and vice versa.

### AGG2 can Complement some but not all *agg1* Mutant Phenotypes

The *agg1 AGG1p::AGG1* self-complementation and *agg1 AGG1p::AGG2* cross-complementation lines were characterized to determine their ability to revert several phenotypes observed in the *agg1-1c* mutant. In most assays, the control *AGG1p::AGG1* construct was able to restore the *agg1-1c* mutant phenotype to wild-type, suggesting that the promoter region used in the constructs was sufficient to drive enough expression in the correct tissues to restore the function of the native AGG1 protein.

*Fusarium oxysporum* is a soil borne fungal pathogen which colonizes the vascular system of plants such as Arabidopsis. Symptoms of infection manifest in yellow chlorotic leaves hence disease progression can be quantified by counting the percentage of chlorotic rosette leaves per plant [69], [70]. As previously reported by Trusov *et al*. [61], *agg1-1c* mutants exhibit hypersensitivity to *F. oxysporum* as evidenced by the faster development of leaf chlorosis, being twice the ratio of wild-type nine days after inoculation (Fig. 2A). The hypersensitivity to *F. oxysporum* was restored to wild-type levels in the self-complementation line (*agg1 AGG1p::AGG1*) (Fig. 2A, P<0.05, one way ANOVA). Similarly, in all three cross-complementation lines assayed the ratios of chlorotic leaves were comparable to wild-type (Fig. 2A, P<0.05, one-way ANOVA), indicating that AGG2 fully rescued the *agg1-1c F. oxysporum* susceptibility phenotype.

It has been proven that Gβ attenuates auxin-induced cell division leading to lateral root proliferation, although it does not directly couple auxin signaling [32], [33]. Initial characterization revealed that the *agg1-1c* mutant also contains a larger number of lateral roots than wild-type. This fact, combined with the stele-specific expression pattern observed for *AGG1* led to the hypothesis that AGG1 combines with AGB1 as a negative regulator of auxin-induced cell division with a possible role in the acropetal auxin stream [61]. Our assays confirmed that 14-days-old mutant *agg1-1c* seedlings produce on average ∼25% more lateral roots and root primordia than wild-type plants (Fig. 2B) (P<0.05, one way ANOVA). The self-complementation line restored the number of lateral roots to wild-type levels. Likewise, all cross-complementation lines rescued the phenotype with the numbers of lateral roots showing no significant differences with wild-type, indicating that AGG2 is able to rescue the lateral root phenotype of *agg1-1c* (Fig. 2B). In addition, *agg1-1c* hypocotyls incubated with exogenous 1-naphthaleneacetic acid (NAA) display increased adventitious root formation [61] (Fig. 2C). Our results confirm this observation and also show that either AGG1 or AGG2 can complement this phenotype returning the number of adventitious roots to wild-type levels. Therefore, for both auxin-related responses, AGG2 can successfully complement AGG1 in Arabidopsis.

A number of studies established the involvement of G proteins in germination [52], [71], [72], [73], [74], [75]. In particular, Gγ subunits have a role in the response to osmotic stress during germination with *agg1-1c* seeds being hypersensitive to mannitol [61]. To determine if AGG2 is able to rescue this *agg1-1c* mutant phenotype, relevant seed lines were sown on a single plate containing media supplemented with 6% D-mannitol. The seeds used in this assay were obtained from simultaneously grown plants to ensure synchronized germination and all the experiments were performed in triplicate. The germination percentage of each line was then scored on each plate and averaged between replicates. Our results confirmed the hypersensitivity to D-mannitol in the *agg1-1c* mutant (Fig. 2D and E). The difference with wild-type was most dramatic on day 8 when 80% of wild-type seeds had germinated, compared to 50% of the *agg1-1c* seeds. Interestingly the *agg1 AGG1p::AGG1* self-complementation line did not completely restore germination levels, showing a small but statistically significant difference with wild-type plants (Fig. 2E). Two of the three cross-complementation lines showed similar germination dynamics to the self-complementation line while the third did not show any restoration of the germination levels, perhaps as a result of expression differences due to transgene positional effects (Fig. 2E).

Early flowering is another of the phenotypic characteristics shown by *agg1-1c* mutants [62]. When we determined the flowering times for wild-type, *agg1-1c* mutants, self- and cross-complementation lines, the early flowering phenotype was clearly observable in the *agg1-1c* mutants and was completely restored to wild-type levels in the self-complementation line (Fig. 2F). In open contrast, none of the cross-complementation lines rescued the early flowering phenotype. A small, but statistically significant increase was observed (P<0.05, one way ANOVA), but it was far from reaching the level observed in the wild-type or self-complementation line.

### AGG1 can Complement some but not all *agg2* Mutant Phenotypes

**Figure 3:**
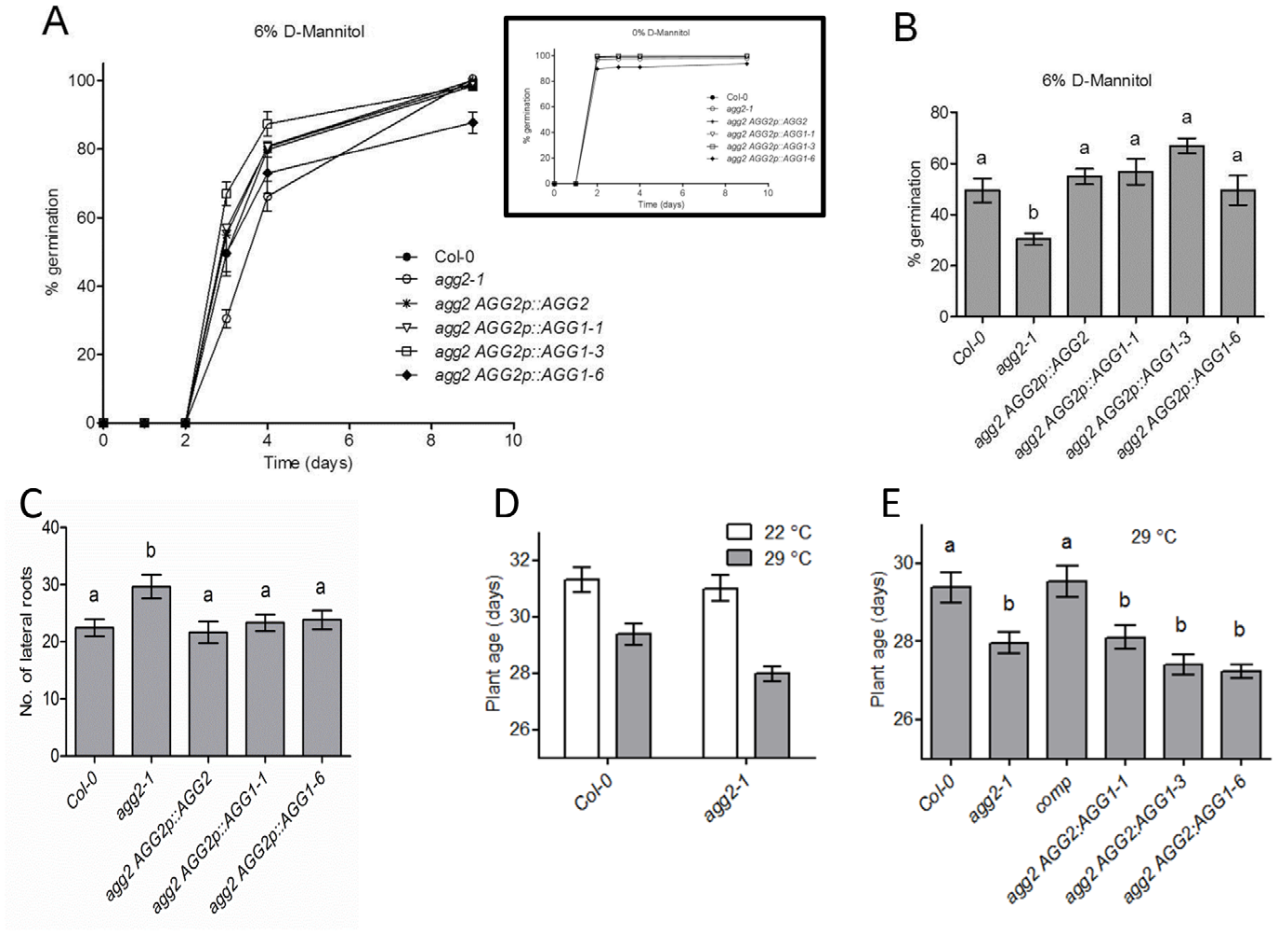
*AGG1* complements some but not all *agg2* mutant phenotypes. (A) Germination dynamics of wild-type, mutant and complementation lines on 0.5x MS supplemented with 6% D-mannitol during 9 days after stratification. Each genotype was analyzed in three replica plates with more than 100 seeds. Insert shows control germination without D-mannitol. (B) Percentage of germinated seeds at day 3 from panel (A) demonstrating highest difference between genotypes. Bars represent average value of three replicates (more than 100 seeds each). Error bars show standard errors. Letters indicate groups with statistically significant differences in seed germination (P<0.05, one-way ANOVA). (C) Total number of lateral roots was scored in two-week-old seedlings grown vertically on 0.5x MS supplemented with 1% sucrose. Bars represent average values ±SE of 15 plants per genotype. Letters indicate groups with statistically significant differences in number of lateral roots (P<0.05, one-way ANOVA). (D, E) *AGG1* failed to complement the *agg2-1* mutant on high temperature-induced flowering. (D) Effect of the *agg2-1* mutation on high temperature-induced flowering. Col-0 and *agg2-1* plants were initially grown at 22°C for two weeks and then divided into two groups: the first group was kept at 22°C, while the second group was transferred to 29°C. Day of inflorescence appearance was recorded for at least 30 plants of each genotype. Bars represent the average number of days from germination till inflorescence appearance ± SE. Letters indicate groups with statistically significant differences (P<0.05, one-way ANOVA). (E) Average number of days from germination till inflorescence appearance in at least 30 plants of each genotype induced at 29°C. Bars represent the average number of days from germination till inflorescence appearance ± SE. Letters indicate groups with statistically significant differences (P<0.05, one-way ANOVA).

As observed for *agg1-1c* mutants, *agg2-1* mutants are also hypersensitive to D-mannitol during germination [62]. When we tested sensitivity to D-mannitol in wild-type, *agg1-1c* mutants, *agg2 AGG2p::AGG2* self-complementation line and three different *agg2 AGG2p::AGG1* cross-complementation lines, the largest differences were observed in day 3 (Fig. 3A). On that time point, the germination percentage of *agg2-1* seeds was significantly lower than wild-type (Fig. 3B; P<0.05, one way ANOVA). The self-complementation line restored germination to wild-type levels as did all three cross-complementation lines (Fig. 3B), suggesting that AGG1 can perform a similar function to AGG2 in the control of osmotic stress. This finding also supports the notion that Gγ1 and Gγ2 have a synergistic role in regulating the osmotic stress response component of germination.

Like *agg1-1c* mutants, *agg2* mutants also produce more lateral roots than wild-type plants. Since the expression of *AGG2* in roots is restricted to the cortex, it was hypothesized that this phenotype was due to defects in basipetal auxin transport or signaling [61]. We therefore investigated whether expressing *AGG1* in the cortex region of *agg2* mutants would restore the root phenotype. As expected, *agg2-1* displayed significantly more lateral roots (including root primordia) than wild-type (P<0.05, one way ANOVA; Fig. 3C). The self- and cross-complementation lines restored the root numbers to wild-type levels indicating that AGG1 and AGG2 are functionally interchangeable in the control of lateral root formation (Fig. 3C).

The early flowering phenotype observed in *agg1-1c* mutants is not observed in *agg2* mutants, at least when grown under long day conditions at 23°C. However, we observed early flowering in *agg2-1* when grown at 29°C compared to wild-type plants (Fig. 3D). To confirm the initial observations, we simultaneously grew at least 30 plants on soil for each of the studied genotypes; i.e. wild-type, *agg2-1* mutant, self-complementation line and three cross-complementation lines. Two trays were initially grown at 23°C under long day regime for each of the lines. After two weeks, one of the trays was moved to 29°C, with similar light conditions and plant age at bolting was recorded for both trays. While no differences in flowering time between wild-type and *agg2-1* were apparent at 23°C, flowering time for *agg2-1* mutants grown at 29°C was significantly shorter than wild-type (Fig. 3E; P<0.05, one way ANOVA). The self-complementation line restored flowering time at high temperature to wild-type levels but none of the three cross-complementation lines successfully rescued the *agg2-1* mutation (Fig. 3E).

## Discussion

Two proteins are functionally redundant when the absence of one can be compensated *in vivo* by the second one. Consequently, a null mutation in one of two redundant proteins will not result in phenotypic alteration (i.e. the mutant will have a wild-type phenotype). Two proteins are functionally interchangeable if they can perform the same biological functions, even if they do not belong to the same species. The Arabidopsis and rice Gγ subunits are obviously not redundant but can be functionally interchangeable if they can complement each other. The fact that *agg1-* and *agg2-*deficient mutants display distinct phenotypic alterations clearly established that AGG1 and AGG2 are not functionally redundant *in planta* and confer specificity to the Gßγ dimer *in vivo* [61]. The first and most obvious explanation for the observed specificity provided by AGG1 and AGG2 is that the Gßγ1 and Gßγ2 dimers activate different sets of effectors in response to diverse signals. However, with futher consideration, the non-overlapping expression patterns observed for *AGG1* and *AGG2* raise the possibility that the observed specificity could be due to the spatiotemporal separation observed for both proteins [61], [62]. It is therefore important to determine whether artificially expressing AGG2 in the same tissues and developmental stages in which AGG1 is normally present will rescue the *agg1* mutant phenotypes. The same logic applies to the reverse. We hypothesized that, given the high degree of similarity between both proteins, AGG1 and AGG2 should be functionally interchangeable.

Our results summarized in Table 1 show that in most cases AGG1 and AGG2 are functionally interchangeable. Four out of five *agg1-1c* phenotypes tested were fully rescued by AGG2, demonstrating that AGG2 is able to functionally replace AGG1 in response to pathogen attack, auxin control of root development and osmotic stress during germination. Similarly, two out of three *agg2-1* phenotypes were rescued by AGG1, proving that AGG1 is able to replace AGG2 in responses linked to auxin in root development and osmotic stress during germination. Collectively, the reciprocal complementation achieved by the two Gγ subunits suggests that both subunits are able to perform many of the same biochemical activities. However, both Gγ subunits are not always functionally interchangeable. AGG2 did not fully complement the *agg1* early flowering phenotype, and AGG1 did not restore the thermosensitive flowering phenotype of *agg2*.

**Table 1:** Summary of Gγ1 and Gγ2 complementation studies.

Mutant background	phenotype	Complementation
*agg1-1c*	Reduced resistance to *Fusarium*	Yes
	Increased adventitious root growth under auxin induction	Yes
	Increased lateral root growth	Yes
	Increased sensitivity to osmotic stress during germination	Yes
	Early flowering time	Partial
*agg2-1*	Increased lateral root growth	Yes
	Increased sensitivity to osmotic stress during germination	Yes
	Heat inducible early flowering time	No

The fact that AGG1 and AGG2 complement each other in pathways responding to osmotic stress, auxin and defense suggests that the Gβγ1 and Gβγ2 dimers activate a number of common effectors, although the identity of those effectors is still unknown. While hypothetical, it is also possible that G-protein involvement could be somewhat indirect resulting from crosstalk among several pathways. Interestingly, there is accumulating evidence suggesting that osmotic stress, defense and auxin pathways modulate each other. Abiotic stress and wounding affect auxin responses [76], [77], [78]. While auxin is implicated in the regulation of plant defense [79], [80]. It is therefore tempting to speculate that Gβγ1 and Gβγ2 may modulate pathways responding to auxin, osmotic stress and pathogen attack at a point, or points of cross talk, using similar signaling mechanisms. This explains why a simplistic repertoire of G proteins functions in such divergent signaling processes.

### Effector Activation by Gß

Alternatively, the fact that AGG1 and AGG2 are functionally interchangeable may indicate that the binding and activation of effectors resides on recognition sites predominantly or exclusively located on the surface of AGB1. Three recent studies revealed that several amino acid residues on AGB1 are essential for effector activation. The first identified acireductone dioxygenase 1 (ARD1) as an AGB1 interactor [81]. Physical interaction was proven in yeast 3-hybrid experiments, while genetic interaction was demonstrated by the rescue of the *agb1-2* short hypocotyl and open apical hook phenotypes of etiolated two-day-old seedlings by *ARD1* overexpression. ARD1 was shown to modulate cell division to control hypocotyl length and AGB1 was able to stimulate ARD1 enzymatic activity *in vitro*. The ability to stimulate ARD1 activity was abolished by several point mutations in AGB1, either single W109, double E248/R25 or triple Q120/T188/R235, suggesting that these residues are essential for ARD1 stimulation. This study proves that AGB1 contains key contact residues for some effectors, such as ARD1.

In the second study, site directed mutagenesis of Arabidopsis AGB1 and the ability of the different mutations to rescue *agb1* phenotypes was tested [82]. Substitution of T65 for alanine rendered AGB1 unable to complement the hypersensitivity of the *agb1* mutant to D-mannitol during germination. In addition, mutation at D250 failed to restore lateral root numbers in the *agb1* mutant to wild-type levels. These observations highlight the importance of individual AGB1 residues in the activation of the effectors involved in osmotic response and lateral root formation. Our results showing that AGG1 and AGG2 are both able to restore D-mannitol sensitivity at germination and lateral root numbers are consistent with the above observations and may indicate that the effectors involved in these two responses form a direct contact with AGB1 residues for activation independently of the AGG subunit attached to AGB1 in the Gßγ dimer.

In a third study, a comparative approach was used to identify a set of residues on the AGB1 surface implicated in protein-protein interfaces [83]. The assumption was that these residues are critical in specific AGB1-effector contacts. Mutation of these residues in combination with genetic complementation assays enabled dissection of the AGB1 protein surface for a variety of ABG1-mediated physiologies (developmental, hormone responses, pathogen defense, and photosynthesis). Interestingly, residues R25 and E248 lie along the AGG-binding tract. Unfortunately, the AGG1-specific differences in flowering reported here were not tested in that study.

### Gγ1 and Gγ2 are not Functionally Interchangeable in the Control of Flowering Time

Our results showed that AGG2 failed to complement the early flowering phenotype of *agg1-1c*, while AGG1 was unable to complement the thermo-sensitive flowering phenotype of *agg2-1*. Although both are flowering time phenotypes, our results suggest that Gßγ1 and Gßγ2 act in separate signaling pathways suggesting that they signal to different downstream effectors and are therefore not functionally interchangeable.

Flowering is a complex process whereby plants go through a transition between vegetative and reproductive phases and is influenced by many environmental factors including photoperiod, temperature, humidity and nutrient availability [84]. Endogenous factors such as carbohydrate reserves and genetic make-up also play a role during the transition phase [85], [86], [87]. Without a comprehensive study of G protein involvement in flowering induction, it is dangerous to speculate as to the specific roles of AGG1 and AGG2 with any confidence. Although speculative, it is interesting to note that G-proteins are implicated in modulating responses to gibberellins (GA) and brassinosteroids (BR), both of which promote flowering [88], [89], [90], [91]. It was recently suggested that G-proteins mediate the cross talk between auxin and BR signaling [91]. AGG1 is clearly implicated in auxin signaling as evidenced by the auxin sensitive traits of *agg1-1c*, opening the door to its involvement in cross talk with BR, therefore having an effect on flowering time through BR-mediated inhibition of FLC the potent flowering suppressor [89]. On the other hand, thermal induction of flowering is dependent on GA, suggesting that the thermosensitive flowering phenotype of *agg2-1* may be due to a role of Gβγ2 in GA signaling [92].

Differential post-translational modification may contribute to the selective functions of AGG1 and AGG2 as has been proven in Cdc42, a GTPase with an important role in the regulation of cell polarity and the actin cytoskeleton [93]. Both AGG1 and AGG2 undergo prenylation, but AGG2 undergoes additional S-acylation, most probably by addition of a palmitoyl group. This second lipid modification was suggested to be the reason AGG2 is able to localize to the membrane more efficiently than AGG1 [94], [95]. Differential membrane affinity plays a major role in mammalian Gγ specialization [96]. It is possible palmitoylation of AGG2 could provide a defining functional difference between the two proteins due to altered membrane affinity. Additionally, lipid moieties are able to form direct contact with effectors and differential lipidation within a protein family can result in conformational variation, allowing different interaction surfaces to be available to different effector subsets [97].

### Conclusions

There is ample proof that the Gβ plays a crucial role in the physical interaction with effectors in Gβγ dimer-mediated signaling in plants and animals [3], [29], [81], [98], [99]. It is also known that, in animal systems, interaction with effectors reside in different Gβ residues [100], [101]. This fact together with the existence of multiple Gβ subunits with divergent sequences can easily provide specificity for the multiple signaling pathways mediated by the Gβγ dimer. However, it is apparent that plants present a very different picture. The openly different phenotypes shown by the AGG1-, AGG2- and AGG3-defficient mutants together with the fact that there are single alpha and beta subunits clearly indicates that γ subunits provide functional selectivity in Gβγ dimer signaling in Arabidopsis. This study investigated the molecular basis for such selectivity in the two prototypical γ subunits, AGG1 and AGG2. AGG3 was not included in the study due to its atypical structural characteristics and strong differences with AGG1 and AGG2, making it highly unlikely to share effectors with the other two γ subunits. Our results show that for some pathways the selectivity is not embedded in their molecular structure, as proven by the ability of AGG2 and AGG1 to complement *agg1* and *agg2* mutants respectively. Effector contact points reside in Gβ and possibly in conserved residues between AGG1 and AGG2. In these cases, specificity is provided by the spatiotemporal differences in AGG1 and AGG2 expression patterns. Nevertheless this is not the case for all phenotypes, implicating that there are some pathways for which signaling specificity is at least partially provided by non-conserved amino acid residues in AGG1 and AGG2. In these cases, specificity is embedded in the molecular structure of AGG1 and AGG2, although differences in expression patterns could also contribute. Contact point/s between effectors and the Gβγ dimer are crucial for effector activation and there are a number of studies that have identified important amino acid residues in Arabidopsis Gβ [81], [82], [102]. The next obvious step is to perform similar mutagenesis studies in AGG1 and AGG2.
